# Advances in Understanding the Initial Steps of Pruritoceptive Itch: How the Itch Hits the Switch

**DOI:** 10.3390/ijms21144883

**Published:** 2020-07-10

**Authors:** Shirin Kahremany, Lukas Hofmann, Arie Gruzman, Guy Cohen

**Affiliations:** 1 Department of Chemistry, Faculty of Exact Sciences, Bar-Ilan University, Ramat-Gan 5290002, Israel; lukas.hofmman@biu.ac.il (L.H.); Aric-Lev.Gruzman@biu.ac.il (A.G.); 2 The Skin Research Institute, The Dead Sea and Arava Science Center, Masada 86910, Israel; guy@adssc.org; 3 Ben-Gurion University of the Negev, Eilat Campus, Eilat 8855630, Israel

**Keywords:** pruritus, itch, receptors, mediator, modulator, GPCR, interleukin, histamine, non-histaminergic, dermal itch

## Abstract

Pruritoceptive (dermal) itch was long considered an accompanying symptom of diseases, a side effect of drug applications, or a temporary sensation induced by invading pruritogens, as produced by the stinging nettle. Due to extensive research in recent years, it was possible to provide detailed insights into the mechanism of itch mediation and modulation. Hence, it became apparent that pruritus is a complex symptom or disease in itself, which requires particular attention to improve patients’ health. Here, we summarize recent findings in pruritoceptive itch, including how this sensation is triggered and modulated by diverse endogenous and exogenous pruritogens and their receptors. A differentiation between mediating pruritogen and modulating pruritogen seems to be of great advantage to understand and decipher the molecular mechanism of itch perception. Only a comprehensive view on itch sensation will provide a solid basis for targeting this long-neglected adverse sensation accompanying numerous diseases and many drug side effects. Finally, we identify critical aspects of itch perception that require future investigation.

## 1. Introduction

Itch is an unpleasant sensation that evokes a desire to scratch, in response to chemical, mechanical, or thermal stimuli [1]. This sensation is a frequent symptom originating from numerous diseases such as dermatological or systemic diseases and neurologic or autoimmune disorders. Eventually, almost one-third of the global population will suffer from itch through the course of their lives [2]. Recent studies revealed that this frequently ignored symptom can have major consequences on physical and emotional health [3,4,5]. Itch is a unique sensory modality in that it is restricted to the skin, mucous membranes, and cornea, as no other tissue or organ is capable of experiencing itch [6]. So far, itch has been the least understood and researched somatosensory modality in the scientific community. Itch has attracted surprisingly little attention until recently despite the advent of the molecular era of biomedical research in the 20th century [7]. Reasons for such low interest and slow progress related to itch are as manifold as the causes for the symptom.

The molecular mechanisms involved in itch sensation are highly complex and remain elusive in most of these diseases. However, from studies in patients and animal models, a large number of controversial mediators, modulators, and receptors responsible for scratching behavior have been identified [8]. The most well-supported distinction between types of itch is that of histaminergic and non-histaminergic itch [9]. These observations have paralleled a much-needed increase of investigation into the mechanisms underlying itch and will eventually lead to new and effective therapeutics [7,10]. The scope of this review is to summarize the current knowledge and recent advances pertaining to the molecular mechanisms of itch, and classify the receptors by mediators and modulators.

## 2. Types of Itch

Itch is classified into four different clinical categories [11,12]. These are neuropathic, systemic, psychogenic, and pruritoceptive [11]. These categories are based on advances in understanding of the peripheral and central origins of itch. In many patients, more than one of these categories can coexist [13]. This review emphasis is placed on pruritoceptive (dermal-generated) itch following molecular mechanisms of pruritus. Neuropathic itch is a result of damage to the central or peripheral nervous system and could occur along the afferent itch pathway that results in a sensation to scratch [14,15]. Systemic itch refers to itch generated from the central nervous system in response to circulating pruritogens, as in cholestasis, or in response to intraspinal morphine [12]. Psychogenic pruritus is defined as itch not related to dermatologic or systemic causes. It can be categorized as a pruritic disease with psychiatric sequelae, a pruritic disease aggravated by psychosocial factors, or a psychiatric disease-causing pruritus [16]. Pruritoceptive itch starts in the skin, usually by an inflammatory or other visible pathological process (e.g., scabies, urticaria) [17]. This type of itch accounts for many cases of clinical pruritus because nearly everything from endogenous mediators to exogenous allergens that invade the skin can induce pruritoceptive itch [18]. As mentioned before, transmission of pruritus can be divided into histaminergic and non-histaminergic [9,19]. Acute pruritus is mediated via both pathways [20,21,22]. In contrast, chronic pruritus is mainly mediated by the non-histaminergic pathway [7]. Further, chronic pruritus can be a seriously debilitating symptom accompanying various cutaneous and systemic disorders [23]. The classification of these two systems originates in the periphery, where the primary afferent nerves express their dedicated receptors [24,25]. Until recently, it was thought that histamine was the final mediator of itch, but clinical observations showed that itch could be initiated without flare by cutaneous electrical or mechanical stimuli, suggesting a histamine independent pathway [26].

**Figure 1:**
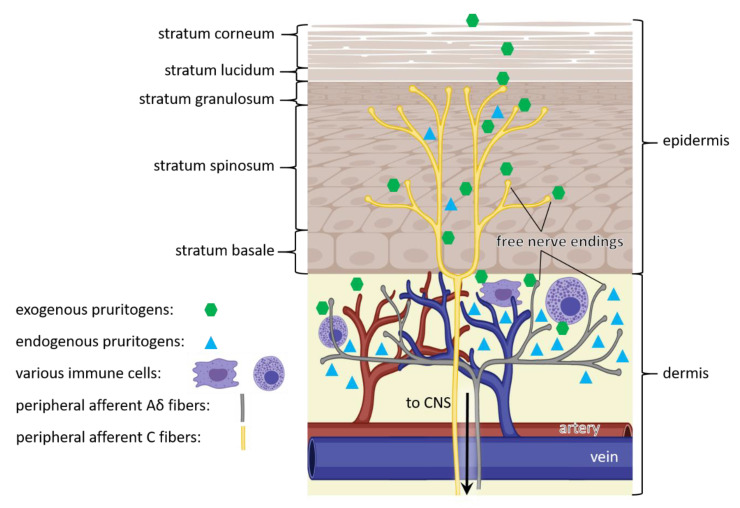
Pathway of itch. Itch sensation is caused by exogenous (green) and endogenous (cyan) pruritogens, that bind to itch receptors in free nerve endings of C fibers within the epidermis (yellow) and Aδ fibers within the dermis (grey). In addition, endogenous pruritogens (cyan) can be produced by epidermal keratinocytes and dermal immune cells (purple). The triggered signal is transmitted through peripheral afferent nerve fibers (yellow and grey) of the peripheral nervous system to the central nervous system (CNS), eventually resulting in itch sensation. (Figure adapted from [28]).

On a molecular level, pruritoceptive itch is initiated when endogenous or exogenous pruritogens interact with itch receptors or pruriceptors (pruritus + receptor). Pruriceptors reside in the membrane of the free nerve endings of peripheral afferent nerve fibers (Figure 1) [27]. These itch-sensory (pruriceptive) nerve fibers are primary sensory neurons, comprised of C fibers (peptidergic and nonpeptidergic) and Aδ fibers [25,28,29,30]. Peptidergic or nonpeptidergic C fibers are unmyelinated and range into the epidermis, responding to itch stimuli, whereas Aδ fibers are lightly myelinated and distributed throughout the dermis, responding to pain and itch stimuli [31,32]. Further, large-scale single-cell RNA sequencing grouped these pruriceptive neurons into three different classes based on the expression of itch receptor types [25,33]. Once these receptors are activated, the signal is transmitted via spinal dorsal horn to the brain [31]. It was revealed, that the transmission of itch sensation by pruriceptive sensory neurons is dependent on the gastrin-releasing peptide receptor (GRPR) within the spinal cord [34], indicating a modulating effect of itch sensation within the central nervous system [1]. For example, it was demonstrated that the μ- and κ-opioid receptors interact with GRPR modifying itch perception in the spinal cord of mice [35,36,37]. More information about itch circuits and its central modulation can be found in the following reviews [1,38,39]. The succeeding paragraphs discuss peripheral receptors followed by their mediators and modulators which are reported to contribute to pruritoceptive itch sensation.

## 3. Itch Mediators, Modulators and their Receptors

**Figure 2:**
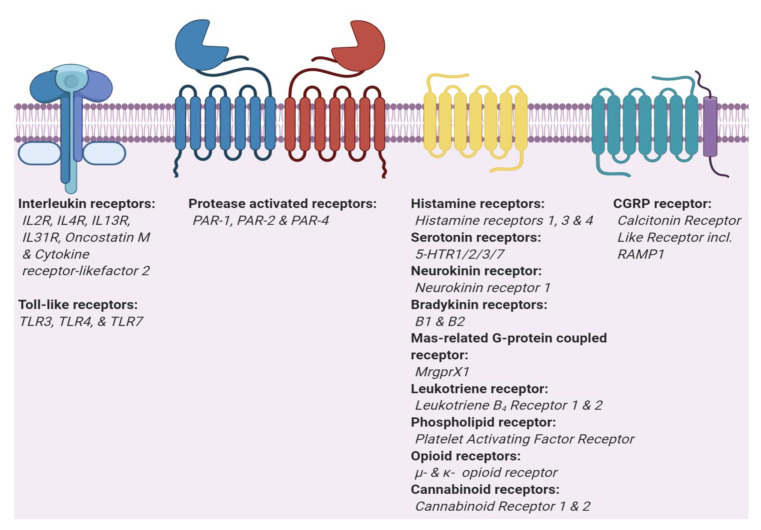
Two main types of itch receptors. Itch receptors can be grouped in two main types, interleukins and G protein-coupled receptor (GPCRs). Toll-like receptors (TLRs) and interleukin receptors (ILRs) are part of the Interleukin-1 Receptor/Toll-like Receptor Superfamily. GPCRs are divided into three sub-types, classic GPCRs, protease activated receptors that dimerize [41,42], and GPCRs which require a receptor activity modifying protein (CGRP).

**Figure 3:**
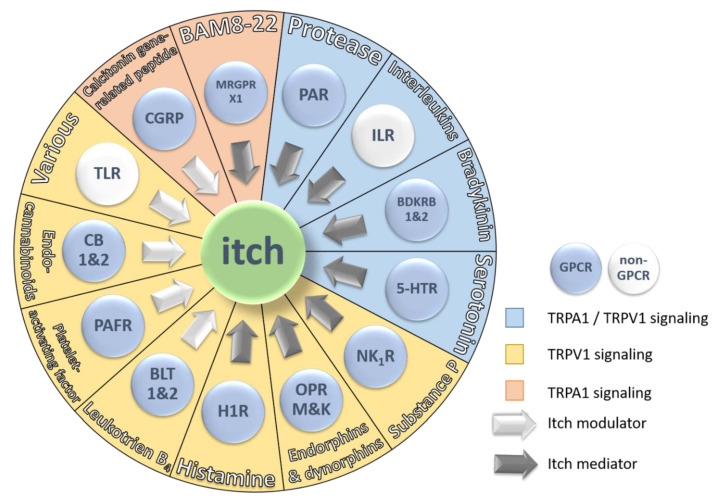
Schematic representation of the 13 major receptor groups involved in itch and their endogenous pruritogens. Each receptor is colored based on two parameters: First, GPCR signaling (blue button) or non-GPCR signaling (white button) and second, ion channel signaling pathway. TRPA1/TRPV1 signaling is represented by a blue sector, TRPV1 signaling is represented by a yellow sector and TRPA1 signaling is represented by an orange sector. The arrows indicate the type of contribution of the receptor and its endogenous pruritogens to itch sensation. White arrows represent itch modulators and grey arrows represent itch mediators.

Pruriceptors localize in the peripheral nervous system and are capable of detecting environmental changes and then transducing this signal to the central nervous system [7]. The majority of itch receptors are members of the class A G protein-coupled receptors (GPCR) (Figure 2). GPCRs are the largest and most diverse group of membrane receptors found in eukaryotes. To date, about 35% of all approved drugs target different classes of GPCRs, suggesting that GPCRs are predestined to be targets to develop drugs relieving or modulating itch sensation in patients suffering from pruritus [40]. Figure 3 represents 13 receptors, their mediators, and modulators involved in itch sensation.

Itch sensation starts with the invasion of pruritogens on the skin. Endogenous or exogenous pruritogens activate receptors which ultimately trigger an itch response (Figure 1). GPCRs, Interleukins, and Toll-like receptors promote the opening of ion channels, namely transient receptor potential vanilloid 1 or 4 (TRPV1/4), transient receptor potential ankyrin 1 (TRPA1), or both to generate action potentials [7,24]. These channels are expressed on all cell types involved in itch signaling, such as keratinocytes, immune, endothelial, and mast cells [43]. Thus, these channels contribute to acute and chronic itch sensation. There are two pathways describing itch stimulation. First, the histaminergic pathway, which activates the TRPV1 channel and second, the nonhistaminergic pathway that activates TRPV1 or TRPA1 (Table 1) [7]. In both pathways, histaminergic and nonhistaminergic, TRPV1/TRPA1 activates NaV1.7, and subsequently, NaV1.7 controls action potentials in neurons [7,24,44].

Here, itch signaling was divided into two classes of stimulants. The first class is endogenous or exogenous mediators, which cause itch by direct activation of pruriceptors, termed itch mediators. While the second class of pruritogens has an indirect effect on itch signaling, these are termed modulators (Table 1 and Figure 3). Receptors activated by such modulators can enhance or reduce itch sensation but do not, or only to a reduced extent, induce itch sensation. It is noteworthy that mediators are capable of potentiating or influencing each other’s effect on itch sensation. Taken together, a mediator can modulate but a modulator cannot mediate itch sensation on its own.

### 3.1. Histamine 1 Receptor

The four histamine receptors (H1-4R) belong to the class A GPCRs and bind to endogenous histamine [45]. H1R is expressed in smooth muscles, and endothelial cells throughout the CNS and cardiovascular system [46,47]. Thus, H1R signaling has an impact on inflammatory processes and circadian rhythm in humans [48,49]. Once activated by histamine, H1R signals through Gi/Go and Gq/G11 with subsequent activation of TRPV1 (Table 2) [50]. H1R shows the lowest potency of all four histamine receptors H3R = H4R > H2R > H1R [46,51]. Albeit it has the lowest potency, the H1R plays a major role in eliciting itch sensation in humans demonstrated by treatment with highly selective H1R antagonists and agonists [52,53,54]. Moreover, recent findings suggest that also H4R and to a lesser degree H3R might have modulatory (additive) effects on pruritus in mice and humans [47,55,56,57]. In conclusion, there is convincing evidence that H1R is responsible for inducing itch sensation in humans. However, more data are required to shed light on the precise role of the other histaminergic receptors in pruritus. Thus far, it seems that H2R does not play a role in pruritus, whereas H3R and H4R solely have a modulatory function in itch sensation.

Histamine has various functions as anaphylaxis mediator, neurotransmitter, and in gastric acid secretion. Histamine is synthesized from histidine by removing the carboxyl group through histidine decarboxylase [58]. Early on it has been shown that administration of histamine produces a triple response of redness, flare and swelling in addition to itch [59]. Moreover, the release of endogenous histamine upon insect bites explained the itching sensation thereafter [60]. The vast amount of literature and research articles on histamine and itch sensation provides a thorough understanding about histaminergic itch sensation and can therefore be considered as the prototypical pruritogen in humans [61].

### 3.2. Serotonin Receptor

Serotonin receptors (5-HTR) belong to the class A GPCRs and can be divided into seven main groups (e.g., 5-HTR1-7) [62]. In total, fourteen 5-HTRs are further subdivided based on sequence, splice variants, specific agonists or antagonists and signal transduction [63]. Given the large group and variety in signal transduction, it seems obvious that 5-HTRs are involved in many biological and neurological processes through the regulation of hormones and neurotransmitters [64,65,66]. Current research in mice demonstrated that 5-HTR1/2/3 and 5-HTR7 are involved in itch mediation, generally application of serotonin induced elevated scratching [67,68,69] (Table 2). In contrast, treatment with 5-HTR2 and 5-HTR3 antagonists did not attenuate scratching behavior in rats, thus contradicting the role of 5-HTR2/3 in pruritus [70]. Interestingly, different strains of rats reacted differently to 5-HT receptor agonists or antagonists as shown by Tian Bin et al. [71]. Aside from the controversies, the role of 5-HTR7 in acute itch seems to be well established. It was demonstrated that activation of 5-HTR7 leads to the opening of TRPA1 and subsequent acute itch sensation [69] (Table 1). Indeed, recent data confirmed that 5-HTR7 signals in an TRPA1-dependent manner, whereas 5-HTR2 acts via an unusual TRPV4 dependent pathway [72] (Table 1). It remains elusive how results obtained in rodents can be applied to humans and thereby answer the role of 5-HT receptors in pruritus. Nevertheless, it was shown that the selective serotonin reuptake inhibitor was capable of reducing pruritus in patients suffering from AD [73,74]. Moreover, it was discovered that 5-HT1A and 5-HT2A were overexpressed in lesional skin compared to healthy patients, indicating a neuromodulator role of serotonin and 5-HT receptors in itch mediation [75]. In addition, recent research provided insight into the role of 5-HT1A receptor, which potentiates itch sensation through gastrin-releasing peptide dependent scratching behavior [76]. To date, it remains controversial if the pruritic influence of 5-HT receptors is fulfilled by their immunomodulatory effect or as a direct itch mediator [74]. Taken together, itch mediated by 5-HT receptors is a complex topic by itself and might be best answered by a dynamic interplay of several 5-HT receptors and their neuroimmunological roles interacting in concert to create the sensation of itch.

Serotonin was discovered in the 1940′s and identified as “tonic” substance from the “serum”, hence serotonin [77]. Serotonin has the rare ability to act as hormone (periphery) and neurotransmitter (CNS) depending on the site of action. In the initial step of its synthesis, L-tryptophan is converted to L-5OH-tryptophan by tryptophan hydroxylase, which is subsequently converted to serotonin by L-amino acid decarboxylase [78]. Humoral Serotonin is responsible for a wide variety of functions in the human body, starting from cardiovascular function, bowel motility, ejaculatory latency, bladder control, platelet aggregation, circadian rhythm, mood, anxiety, appetite, temperature just to name a few [65]. It was also demonstrated that the application of serotonin potentiates and induces itch sensation in mice [69,79]. Thereby, serotonin contributes to vasodilation, inflammation, immunomodulation and pruritogenic effects by interacting with their receptor in the skin [74]. Thus, explaining pruritogenic effects elicited by serotonin without considering the synergistic effects of inflammation, vasodilation, and immunomodulation would be insufficient. It remains a tremendous and complex task to decipher the individual contribution of each 5-HT receptor and serotonin to acute itch sensation in human patients.

### 3.3. Protease-Activated Receptors

Protease-activated receptors (PAR) belong to the class A GPCRs and are unique from most other GPCRs in their mechanism of activation. They are activated by proteolytic cleavage of their own extracellular N terminus [80,81,82]. Once activated, PARs signal through Gi/Go and G12/G13, whereas PAR1 is also capable of signaling through Gq/G11 heterotrimeric G proteins [51] (Table 2). This family is comprised of four members, PAR1, PAR2, PAR3, and PAR4, which are involved in a variety of physiological functions ranging from hemostasis, inflammation to cell differentiation and proliferation [83,84]. Studies demonstrated that activation of PAR1, PAR2, and PAR4 can induce non-histaminergic itch. [38,85]

There are various endogenous and exogenous mediators that have been shown to induce itch sensations. These mediators belong to the protease family, examples are cathepsin S, mucunain, and tryptase [86,87,88]. Cathepsin S is an endogenous lysosome protease that was shown to evoke itch and activate PAR2 and PAR4. [89] Mucunain is an exogenous cysteine protease that was shown to also activate PAR2 and PAR4. Mucunain is the active component of cowhage (Mucuna pruriens), a plant found in tropical areas. It was reported that this compound elicits pruritus without the urticarial response associated with histamine suggesting that mucunain could identify a non-histaminergic pathway of pruritus [80,90]. Other plant-derived cysteine proteases such as bromelain, ficain, and papain activated PAR2 and PAR4 in HeLa cells [91]. Given that these proteases act via the same activation mechanism as mucunain, would suggest a histamine independent mechanism of action. Aside from plants there are many other exogenous proteases originating from bacteria, amoebae, insects, fungi, and reptiles activating members of the PAR family [83,92,93]. Nonetheless, the detailed mechanism of how and if these exogenous proteases elicit an itch sensation exclusively through PARs remains to be answered.

PARs are known to form homo- or heterodimer with other members of the PAR family [42,94,95,96]. How this dimerization impacts and modulates itch sensation is still under investigation. Obviously, the dimerization interface of PARs provides an additional target site to reduce or modulate itch sensation triggered by protease mediators. Additionally, activation of PAR1 and PAR2 recruit trimeric G proteins (Gi, Gq, or G12/G13) whereas PAR4 signals, through Gq or G12/G13. Gq activation, stimulate further downstream processes including TRPV1 and TRPV3 [42,88,97,98] (Table 1 and Table 2). If all members of PAR cause itch sensation involving TRPV1 activation or if other G proteins, biased signaling, and additional receptors are engaged remains elusive.

### 3.4. Neurokinin Receptors

There are three types of neurokinin receptors: NK1R, NK2R, and NK3R. All belong to the class A GPCRs. Moreover, all three members bind to peptide ligands and signal via Gq/G11, whereas NK1R and NK2R are also capable of Gs signaling [51] (Table 2). Among NK receptors, NK1R is mainly involved in itch because of its preferred ligand substance P (SP) [99,100]. Interaction with SP leads to activation of phospholipase CB (PLCB), and results in a transient increase in intracellular inositol 1,4,5 triphosphate (IP3) diacyl-glycerol and increased cytosolic calcium concentration [101]. NK1R is expressed by sensory nerve endings, mast cells, keratinocytes, and fibroblasts and is highly abundant in the central nervous system [100,102]. Moreover, it was reported that NK1R is overexpressed across multiple chronic itch-induced conditions [103]. Thus, treatment of patients suffering from chronic pruritus reported reduced itch sensation after various NK1R antagonist regimens [99,104,105]. Recent research suggested an interplay between opioid receptors and NK1R because of their co-localization. There is growing evidence that opioid receptors might modulate SP release and that NK1R is involved in side effects of opioids [106]. Therefore, it is possible that the interplay between opioid receptors and NK1R might also modulate the signaling of itch sensation through SP.

SP is a neuropeptide and consists of eleven amino acid residues [107,108]. This undecapeptide belongs to the largest known peptide group, the tachykinin peptide hormone family (locus on the TAC1 gene) [109]. It was shown that overexpressing SP nerve fibers are augmented in human skin suffering from chronic itch [110]. Thus, SP remains an interesting neuropeptide with regard to the mechanisms of itch sensation. Even though SP binds to NK1R and MRGPRX2 only NK1R is involved in itch sensation since the effect was reduced by administering NK1R specific antagonist which exclusively binds NK1R but not MRGPRX2 [111]. Furthermore, SP is known to induce expression of leukotriene B4 which in turn causes itch sensation through leukotriene receptors [112]. Thus, SP induced itch sensation might be evoked by induced expression of leukotriene B4. Moreover, histamine and SP induced itch was almost completely suppressed by antihistamines, whereas bradykinin- and serotonin-induced itch was not. Suggesting that SP is a histamine-dependent pruritogen [113]. The complex interplay between endogenous pruritogens demonstrates that further research is urgently required to fully understand the role of each receptor and mediator in the orchestra of itch sensation.

### 3.5. Bradykinin Receptors

As the majority of pruritogenic receptors, also bradykinin receptors BDKRB1 and BDKRB2 belong to the class A GPCRs. BDKRB1 and BDKRB2 signal through trimeric G proteins Gi/Go, Gq/G11 and Gs, Gi/Go, Gq/G11, respectively [114] (Table 2). Another major difference between BDKRB1 and BDKRB2 are the expression levels. While BDKRB2 is constitutively expressed, BDKRB1 is solely expressed in traumatic or inflammatory conditions [115]. Even though bradykinin (BDK) is known to activate both receptors the role of the two receptors in BDK induced itch remains enigmatic. Whether both receptors BDKRB1 and BDKRB2 induce itch sensation to a similar extend, if they interact via a complex interplay or via identical pathways is part of current research [116]. Previous studies demonstrated that a BDKRB2 antagonist induces itch sensation and this sensation is increased in combination with BDK [116]. Given that BDKRB1 causes itch sensation upon BDK application, an obvious explanation would be that BDKRB1 is activated and BDKRB2 inactivated, causing the synergistic effect of BDK and BDKRB2 antagonist. Conversely, another study concluded that itch induced by sodium deoxycholic acid is reduced upon administration of BDKRB2 antagonists [117]. Activation of BDKRB1 mediates the alloknesis response in complete Freund’s adjuvant (CFA)-inflamed mice [116]. Investigations showed that activation of BDKRB1 receptor with BDK caused histaminergic independent itch sensation in inflamed tissue [113,116]. In addition, it was demonstrated that itch related behavior was modulated but not entirely depleted upon prior application of histamine [118,119]. Thus, demonstrating histaminergic independent itch sensation induced by BDK [113]. Further, it was also shown that the administration of BDK causes elevated concentrations of CGRP, substance P, and prostaglandin E2, all of them are known to induce itch sensation [120]. Taken together, these findings illustrate the controversial understanding of itch mediated by the two BDK receptors. The common denominator of these studies is related to the fact that both receptors are involved in itch sensation but through different pathways. Both of these pathways triggered by BDKRB1 and BDKRB2 require further investigation to fully understand the interplay between the BDK receptors BDKRB1, BDKRB2, and histaminergic or non-histaminergic pruritus.

Aside from the controversies about the receptor signaling, it is well established that BDKRB1 and BDKRB2 have the shared ligand bradykinin. BDK is a nonapeptide hormone (sequence RPPGFSPFR) originating from the kinin-kallikrein system [121]. Thereby, the precursor kininogen is enzymatically cleaved to produce BDK by kallikrein which itself is originating from the precursor prekallikrein [122]. Kinins, which are produced by the action of kallikrein enzymes, are blood-derived local-acting peptides that were shown to have broad effects. The nonapeptide is generated mainly during pathophysiologic conditions such as inflammation, trauma, burns, shock, and allergy, thus causing skin disorders in humans and mice under chronic inflammation conditions [123]. In addition, it was shown that an injection of highly diluted BDK (1: 100 Mio) is sufficient to elicit a physiological itch response, which renders BDK a highly potent pruritogen [124].

### 3.6. Calcitonin Gene-Related Peptide Type 1 Receptor

The calcitonin gene-related peptide type 1 receptor (CALCRL) is an atypical member of the class B1 GPCRs required to carry a modification in order to be functional. This modification is called receptor activity-modifying protein (RAMP1), which results in a heterodimeric receptor entity required for trafficking and receptor activity [125,126,127]. This heterodimeric receptor is called calcitonin gene related peptide (CGRP) receptor and is capable of signaling via Gs heterotrimeric G proteins [128] (Table 2). CGRP receptors are translated throughout the body and are involved in a variety of physiological processes of which mediation of migraine is of particular interest [129]. Nevertheless, it was shown that ablation of CGRP resulted in attenuated itch, suggesting a modulating effect of CGRP receptors [130]. Moreover, it was demonstrated that neurons lacking expression of CGRP resulted in reduced capsaicin/heat associated itch response in mice [131]. Taken together, CGRP receptors are receptive to a variety of physical stressors ranging from heat, cold, mechanical stress to itch. Therefore, it is topic of current research to elucidate the complex interplay of CGRP signals with other neurotransmitters to elicit the above-mentioned sensation influenced by CGRP and its receptor.

CGRP a member of the calcitonin peptide family, consisting of two members αCGRP and βCGRP which both are 37 amino acids long and share 94% identity [132,133]. CGRP is the product of transcription, translation, and post-translational modification of the calcitonin gene. Thereby, the two mature CGRP result from splicing, post-translational modification, and protease cleavage [134]. These mature isoforms of the neurotransmitters are then stored in vesicles at the sensory nerve terminals which are frequently released together with substance P [135]. Given the interplay between substance P and CGRP, the simplistic view of CGRP induced itch fails to explain the synergistic outcome of the two endogenous modulators. Thus, only a comprehensive view on itch mediators as published by Rogoz et al. provides a profound understanding of CGRP modulated itch sensation [130]. In addition, recent studies have reported that prurigo nodularis, a disorder accompanied by intensive pruritus, is closely associated with increased dermal levels of CGRP and SP [136]. Altogether, itch sensation modulated or induced by CGRP is a complex subject involving various transmitters leading to synergistic effects. Thus, only a combinatorial treatment against pruritus will be effective.

### 3.7. Mas-related GPR Family Member X1 (MRGPRX1)

MRGPRX1 belongs to the class A orphan GPCRs, albeit lacking the conventional Cys-Cys disulfide bridge and ionic-lock, which are found in prototypical class A GPCRs [137]. Also, MRGPRX1 is a member of the MRGPR family, which comprises receptor genes found in mice, rats, and humans [138,139]. The MRGPR family consists of eleven subfamilies with about 50 GPCRs in total, seven subfamilies and ten GPCRs thereof were detected in humans [140]. It was found that MRGPR is exclusively expressed in dorsal root ganglion neurons and these sensory neurons are responsible for detection of painful stimuli [140,141]. Interestingly, these neurons containing MRGPRs lack the expression of TRPV1, substance P, and partly CGRP in mice but not in humans [141,142]. The substantial difference in expression patterns of itch modulators and mediators such as substance P, CGRP and TRPV1 between mice and humans obscures a direct transfer of results about MRGPR mediated pruritus [141]. In humans, MRGPRX1 receptor is activated by bovine adrenal medulla peptide (8–22) (BAM8-22), compound 16, and chloroquine. Activated MRGPRX1 signals through the Gq/G11 pathway resulting in stimulation of phospholipase C [21,143,144,145] (Table 2). It was shown in mice that activation of MRGPRA3 and MRGPRC11 (MRGPRX1 mouse homolog) with BAM8-22 or chloroquine ultimately leads to activation of TRPA1 channel which elicits itch sensation [146] (Table 1). Chloroquine is commonly used as an anti-malaria therapeutic and found recent popularity during the COVID-19 outbreak. In addition, pruritus is one of the most common side effects in chloroquine treatment, which also shows poor response to systemic antihistamine pre-treatment [147,148,149]. The case studies mentioned here together with the human patient trial of BAM8-22 provide strong evidence for the role of MRGPRX1 in pruritus [150]. However, effective concentrations of endogenous agonists for MRGPRX1 are rarely reached, thus the question of the role of MRGPRX1 remains open. One hypothesis for the role of MRGPRX1 is related to the fact that this receptor is predominantly activated by warning of toxic or harmful substances invading the organism [140]. Despite strong evidence for the itch inducing role of MRGPRX1 in humans, it might not be the target of choice for treating pruritus or itch related diseases, except if induced by chloroquine or any other known exogenous agonists mentioned above.

Bovine adrenal medulla (BAM) peptides are peptides secreted from the adrenal gland derived from cleavage products of the pro-encephalin A gene [144,151]. It was shown that the first seven amino acids of BAM22 do not contribute to the binding to MRGPRX1, whereas these initial amino acids are crucial in binding to opioid receptors [152]. It was shown that application of BAM8-22 induced itch sensation without wheal or flare in a histamine independent manner, but this was not induced by BAM8-18 [153]. In addition, it was shown that MRGPR can be activated by the peptide SLIGRL, which is released upon activation of PAR2 in mice [154]. Moreover, the truncated version SLIGR lacking the leucine amino acid residue is capable of activating PAR2 but the mouse MRGPR lacks an itch response [154]. This controversy indicates that itch mediated by SLIGRL might be evoked by the activation of MRGPRX1 and not PAR2. Thorough research is required to precisely describe the role of the two receptors and their impact in itch mediation.

### 3.8. Leukotriene Receptors

Leukotriene receptors are class A GPCRs that bind and are activated by leukotrienes (LT). They include cysteinyl leukotriene receptor 1 and 2 that are activated by LTC4, LTD4, and LTE4 [155]. Additionally, leukotriene B4 receptor 1 (BLT1) and leukotriene B4 receptor 2 (BLT2) are activated by leukotriene B4 (LTB4). BLT1 is signaling through Gi/Go and Gq/G11, whereas BLT2 mainly signals through Gi/Go [51] (Table 2). Andoh et al. found that scratching behavior of mice could be induced after the injection of LTB4 into mice skin. It was found that the levels of LTB4 were significantly elevated in AD and psoriatic lesions which were usually accompanied with pruritus [156]. These studies in mice showed a connection between LTB4 and itch sensation [157,158]. Furthermore, the application of BLT antagonist ONO-4057 suppressed the itch sensation, indicating involvement of BLT receptors in pruritus in mice [159]. However, LTB4 induced itch could not be recapitulated in healthy humans, and only wheal and flare response was observed upon LTB4 application [160,161]. Thus, it is highly controversial if leukotrienes are directly involved in itch sensation. Nevertheless, it is of common understanding that leukotrienes play a crucial role in inflammation but in contrast play at most a modulatory role in itch sensation [155].

The origin of LTs is found in the universal precursor arachidonic acid [162]. This subfamily of molecules belongs to eicosanoids signaling pathways which are responsible for diverse physiological and pathological functions [163]. LTB4 is known to bind both receptors; BLT1 with high affinity and BLT2 with low affinity [155,164]. To date, extensive research showed a major contribution of LTB4 in inflammatory diseases but clear evidence for an explicit role in itch sensation is still lacking.

### 3.9. Platelet-Activating Factor Receptor (PAFR)

PAFR belongs also to the class A GPCR family as the majority of receptors involved in itch sensation. PAFR is mainly localized in the plasma and nucleus membrane of inflammatory cells, immune cells and cells of the hemostatic system [165]. Thereby, phospholipid ligands bind to PAFR and induce conformational changes, which in turn recruit various heterotrimeric G proteins, such as Gs, Gi/Go and Gq/G11 [114] (Table 2). This intracellular signal cascade leads to an amplification of inflammatory and thrombotic events such as synthesis of interleukins and activation of target cells [165]. Research administering a combination of platelet-activating factor (PAF) and the H1R antihistamine mepyramin resulted in reduction of pruritic effects. Similar results were observed in histamine-depleted skin [166]. Additionally, it was shown that intradermal administration of PAF caused itch sensation through histamine, released from mast cells via neurogenic activation [167,168]. Yet, a detailed mechanism of how PAF activates these peptidergic neurons is still under investigation.

Platelet-activating factor (PAF) is produced through two different enzymatic pathways [169]. The first pathway comprises a remodeling pathway substituting an acetyl residue for the fatty acyl residue of membrane derived phospholipids. The second pathway describes a *de novo* synthesis which involves the two enzymes phospholipase A2 and lyso-PAF acetyltransferase thereby synthesizing PAF from phosphocholine and an alkyl acetyl glycerol rest [170]. Additional information regarding the synthesis and homeostasis of PAF and other lipid mediators can be found in the excellent review by Prescott et al. [169]. PAF has a variety of physiological and pathophysiological effects. It acts as an important mediator and activator in anaphylaxis, inflammation, platelet aggregation and degranulation, and leukocyte chemotaxis. Normally, PAF is produced in low quantities by various cells (e.g., platelets, neutrophils, macrophages, endothelial cells, and monocytes), but it emerges in larger quantities from inflammatory cells in response to specific stimulators. Through specific receptors and a series of signal transduction systems, PAF works to induce diverse biochemical responses. It has been demonstrated that PAF initially evokes an inflammatory response in allergic reactions in the skin of mammals and humans. Further, prolonged exposure of PAF antagonist resulted in a desensitization of the said antagonist [171]. This mechanism indicates an upregulation of PAFR expression or increased receptor activity after acquired pharmacodynamical tolerance to compensate for lost sensitivity. Whether receptor sensitivity or receptor density was increased or if a third mechanism is responsible for the acquired tolerance remains the subject of future studies.

### 3.10. Opioid Receptors

Opioid receptors belong to class A GPCRs as the majority of itch mediating receptors. It was previously shown that out of the four different opioid receptors only μ- receptor (OPRM) as heterodimer with gastrin-releasing peptide receptor (GRPR) and κ- receptor (OPRK) mediate itch sensation [37,172,173,174,175]. Thereby, OPRM and OPRK both signal through Gi/Go heterotrimeric G proteins [176] (Table 2). In addition, recent research showed that GRPR in the CNS is required for morphine induced itch sensation [35]. Also, it was shown that up to ten percent of patients treated systemically with opioids (morphine) developed pruritus [177]. It is a topic of current research whether expression levels of OPRM in the skin are altered in patients with pruritus. It was recently demonstrated that OPRK expression levels were indeed downregulated, whereas OPRM levels remain unchanged in patients suffering from psoriatic itch [178,179]. Thus, it can be concluded that patients suffering from itch might show an imbalance of epidermal opioid receptors being the cause or result of said sensation [180]. Various OPRM antagonists were able to decrease morphine induced itch sensation in human trials [181,182]. Similar effects were reported when OPRK agonists were applied to patients suffering from pruritus [183]. Taken together, there is significant evidence that both OPRM and OPRK receptors or imbalanced expression levels of these receptors are involved in pruritus and itch sensation. Of note, it was shown that both OPRM antagonists and OPRK agonists are able to relieve symptoms of opioid induced itch sensation.

Dynorphin is the endogenous OPRK agonist and an opioid peptide derived from cleavage of prodynorphin by proprotein convertase 2 in the nervous system [184]. In agreement with the OPRK agonist treatment mentioned above, it was shown that the expression and presence of dynorphin inhibited itch sensation in mice [185]. Endorphins are a group of OPRM agonists and are comprised of three endogenous opioid peptides, which are produced and stored in the pituitary gland [186]. It was shown that the plasma levels of β-endorphins are elevated in patients suffering from prurigo [187]. Thus, it can be hypothesized that imbalanced endorphin levels may contribute to pruritus. How endorphins and dynorphins act in patients suffering from pruritus is under investigation. However, there is clear evidence showing the role of these opioid peptides in itch sensation but the effect of imbalanced expression and distribution in pruritus remains to be answered.

### 3.11. Cannabinoid Receptors

Cannabinoid receptors are comprised of cannabinoid receptor 1 (CB1) and cannabinoid receptor 2 (CB2), both belong to the class A GPCRs [188]. The two receptors share 44% identity and signal through Gi/Go heterotrimeric G proteins [189] (Table 2). CB1 is mainly distributed in the central nervous system, while CB2 is distributed in the peripheral tissues mainly in immune and to a lesser degree in neuronal cells [189]. Several studies showed that the topical application of cannabinoid derivatives relieve itch sensation in patients suffering from pruritus [190,191,192]. Moreover, histamine-induced itch was attenuated by CB agonist [193]. These findings clearly demonstrate the involvement of CB receptors in itch sensation. In addition, it was shown that CB1 and TRPV1 receptors are co-localized in primary afferent C-fibers [194]. This co-localization is of particular interest, since TRPV1 channels are involved in histaminergic itch sensation, thus representing the bottleneck of histaminergic evoked itch [195,196,197]. The close interplay between TRPV1 and CB receptors render cannabinoids a preferred substance to counteract histaminergic involved itch sensation. In conclusion, CB receptors might not induce itch sensation but are potent modulators of pruritus.

Endocannabinoids (eCB) belong to derivatives of arachidonic acid and are comprised of N-arachidonoylethanolamide (AEA) and 2-Arachidonoylglycerol (2-AG). These two arachidonate-based lipids are synthesized from arachidonic acid by fatty acid amide hydrolase and monoacylglycerol lipase [198,199]. Together with the two receptors CB1 and CB2 they comprise the so-called endocannabinoid system [200]. Thereby, AEA binds with higher affinity to CB1 and to a lesser degree to CB2, whereas 2-AG binds to both receptors with equal preference [201]. It is well established that the endocannabinoid system is involved in a variety of processes including mood, memory, sleep, appetite, and fertility [202]. In animal studies, it was found that cannabinoid (CB) could induce release of 13- endorphins by binding to their receptor relieving pain and alleviate histamine-induced itching. Furthermore, the endocannabinoid AEA is known to activate TRPV1 and thus release of CGRP [203] (Table 1 and Figure 3). In addition, recent research showed that various cannabinoids are capable of acting on a variety of TRP channels [204]. Thus, it might be possible that AEA- and CB- induced itch sensation is caused by the release of CGRP and SP through activation of TRPV but not by directly interacting with CB receptors (Table 1). These results indicate that eCB, including CB1 and CB2, may be involved in the regulation of pain and pruritus as modulators but not as pruritogens.

### 3.12. Interleukin-1 Receptor/Toll-like Receptor Superfamily

#### 3.12.1. Interleukin Receptors

Interleukin receptors belonging to the cytokine family are playing an important role in the underlying mechanism of itch [205]. Interleukin receptors are activated by cytokines which are released from leukocytes. Herein, we focus on itch related cytokine receptors IL2R, IL4R, IL13R, IL31R, Oncostatin M receptor, and cytokine receptor like factor 2 heterodimerized with IL7Rα.

IL2R exists as combinations of three different proteins, namely IL2Rα, IL2Rβ, and IL2Rγ, and these three proteins together form the high affinity receptor of IL-2 [206]. Once IL2R is activated by IL-2 it propagates the signal via two tyrosine kinases JAK1 and JAK3 associated with IL2Rβ and IL2Rγ respectively (Table 2). Activation of these two kinases triggers the MAPK and PI-3K kinase pathways [207,208,209]. A study carried out on 30 patients suffering from pruritus showed increased IL-2 levels compared to healthy individuals [210]. Furthermore, application of IL-2 showed a low but immediate pruritogenic effect upon subcutaneous administration [211]. These results demonstrate the ability of IL-2 to induce itch sensation albeit to a moderate extent.

IL-2, the endogenous ligand of IL2R, is a 153 amino acid long protein belonging to the hematopoietin family and is produced by CD4 T lymphocytes upon induced cell differentiation [212,213,214]. A study carried out on thirty patients suffering from uremic pruritus and healthy control showed increased levels of IL-2 between patients and control but no correlation among patients suffering from pruritus and without [210]. Further, 10–29% of patients treated with Aldesleukin (IL-2) reported itching as a side effect of the treatment [215]. Altogether, more research is required to decipher the precise role of IL-2 in pruritus or mediation of itch sensation. Thus far, it seems plausible that IL-2 plays a modulatory role in itch sensation but does not necessarily act as pruritogen in humans.

Unlike IL2R, IL4R can consist of two different heterodimers IL4Rα combined with either γc or IL13Rα1, propagating the signal of both IL-4 and IL13R or only IL13R, respectively [216,217,218,219]. Binding of either IL-4 or IL13R results in activation of STAT6 through the JAK1, JAK3 or Tyk2, JAK2 pathways, depending on cell type and receptor heterodimer [220,221] (Table 2). A recent study revealed the pruritogenic effect of IL-4 and IL13R administered to mice. They were able to show an additive effect while administering IL-4 and IL13R in combination [222]. IL-4 and IL13R alone did not evoke itch response in humans, thus these findings in mice could not be recapitulated [223]. Albeit, administration of histamine in combination with IL-4 resulted in increased scratching behavior, indicating a modulatory role of IL-4 in itch responses [223]. These findings indicate that IL-4 might be an endogenous modulator of pruritus but how and if IL-4 evokes an itching sensation in humans remains to be answered.

IL-4 is a pleiotropic cytokine of 153 amino acid residues and is predominantly expressed in mast cells and T cells [224]. AD patients treated with dupilumab, a monoclonal antibody blocking IL4Rα, showed amelioration of their conditions [225,226,227]. Furthermore, a study in mice showed that deletion of IL4Rα sensitizes neurons to other pruritogens and that IL4Rα is required to elicit a chronic itch sensation in AD-like skin inflammation. The same study also showed that JAK1 inhibition in patients with CIP reduces pruritus [223]. Thus, there is supporting evidence showing that IL-4 does not act as acute pruritogen but merely modulates itch sensation by increasing sensitivity to other pruritogens.

IL13R consists of a heterodimer similar to IL4R. The subunits IL13Rα (which also forms one heterodimer receptor of IL4R) and IL13Rα1 compose the functional receptor IL13R [218]. It was shown that IL13R binds to IL13R with high affinity of 10–15 M, allowing for exclusive signaling through IL13R and not IL-4, despite their shared receptor moiety [228]. Once activated, IL13R signals through JAK1 and Tyk2 and STAT6 activation [229] (Table 2). As mentioned above (see IL4R), the research on IL-4 and IL13R showed that IL13R caused scratching behavior in mice [222]. In addition, research carried out on mice suffering on IL13R induced topic dermatitis showed that additional IL13R administration stimulated TRPA1 expression, which is known for its role in pruritus [230]. Thus, it is debatable if IL13R is a pruritogen or if IL13R modulates itch sensation by enhancing the allergic responses of sensory neurons.

IL13R is as IL-4 a pleiotropic cytokine of 146 amino acids, and shares about 30% sequence homology with IL-4 [231,232]. IL13R is expressed by TH2 helper cells, mast cells, basophils, and eosinophiles [233,234]. Aside from the close homology to IL-4, IL13R also activates similar responses as IL-4, partially due to the shared receptor subunit IL13Rα1 [218]. IL13R induces proliferation and immunoglobulin E synthesis in human B cells, this might suggest that IL13R impacts allergic reactions and anti-inflammatory processes [235]. It was shown that mice suffering from AD induced by IL13R, had elevated expression levels of TRPA1, which is one cause of chronic itch in AD [230]. In agreement with this finding, it was published that IL-4 and IL13R elicit scratching behavior in mice [222]. Similar experiments in humans are still lacking. Thus, it is still under investigation whether IL13R is an immediate pruritogen or if the described sensation in mice is due to the allergic or anti-inflammatory role of IL13R.

IL-31 receptor (IL31R) is a heterodimeric receptor consisting of IL-31 receptor A (IL31RA) and oncostatin M receptor (OSMR) [236]. OSMR increases the affinity of IL31R towards IL-31 and activates PI3K/AKT and MAPK signaling pathways. In addition, IL31RA signals through JAK1/2 which subsequently activate STAT1/3/5 upon binding to IL-31 [236,237,238] (Table 2). IL31R are expressed among others in keratinocytes, epithelial cells, and by DRG pruriceptors [238,239]. Immunohistochemical analyses revealed that IL31R protein levels seem to be increased in patients with AD [239,240]. In addition, it was shown that the IL31R is co-expressed with TRPV1 and TRPA1 in sensory nerves which mediate T cell induced itch [241]. Aside of AD, IL31R overexpression and elevated IL-31 levels are involved in various diseases such as allergic contact dermatitis, psoriasis, bullous pemphigoid, chronic spontaneous urticarial, dermatomyositis, bowel disease and airway hypersensitivity [236,242]. Given the significant evidence of the connection between pruritus and prevalence of IL31R, it is beyond doubt that IL31R is the cytokine receptor responsible for transmitting itch sensation in humans suffering from pruritus, thus providing an excellent target for the treatment of pruritus and IL31R related diseases.

IL-31 is 164 amino acid long and belongs to the interleukin 6 family of cytokines [237]. IL-31 is preferably expressed in activated helper T cells, in particular TH2 helper cells, mast cells, macrophages, dendritic cells and eosinophils comparable to IL13R [238,242]. Also, IL-31 plays an important role in the homeostasis of the skin, airway, and intestinal epithelia, in general IL-31 is crucial for an intact innate or adaptive immunity in tissues with direct contact to the environment [236,237]. It was shown that levels of IL-31 were significantly elevated in pruritic lesional skin of patients with AD compared to nonpruritic lesional skin of patients with psoriasis [243]. Similar findings were confirmed in mice, where elevated IL-31 levels lead to pruritus, alopecia, and skin lesions [238,242,244,245]. Moreover, it was shown that IL-31 is overexpressed in patients suffering from asthma [246]. Altogether, there is strong evidence that IL-31 induces pruritus and plays a crucial role in autoimmune skin diseases. Future research is required to answer the details of how IL-31 induces pruritus and causes atopic dermatitis.

Thymic stromal lymphopoietin (TSLP) receptor (TSLPR) is a heterodimeric receptor consisting of IL7Rα and cytokine receptor-like factor 2 [247]. TSLPR is mainly found in immune cell types such as monocytes, T cells, B cells, mast cells NKT cells, dendritic cells, and tissues from heart, skeletal, muscle, kidney, and liver [248,249,250]. TSLPR is activated upon binding of TSLP, thus signaling as other interleukin receptors through activation of JAK1/2 and subsequent activation of STAT1/3/4/5/6 [251,252,253] (Table 2). Remarkably, it was shown that TSLP is able to induce itch sensation by directly activating TRPA1-positive neurons. Still, the study demonstrated that both receptors TSLPR and TRPA1 are required to induce itch sensation upon TLSP administration [254,255]. Taking together, TSLPR plays a crucial role in itch mediation while the sensation may be modulated by direct activation of TRPA1. Future research will provide more insights into the orchestration of itch sensation by TLSPR and additional receptors activated by TLSP.

TSLP exists in two isoforms as a steadily expressed short form and as a long alternative splice variant which is expressed upon inflammation [256,257]. The short isform counts 63 amino acids, whereas the long isoform consists of 159 amino acid residues [258]. TSLP, a pleiotropic cytokine, belongs to the IL-2 cytokine family and is a paralog of IL-7 [248]. Moreover, TSLP is mainly expressed in lung and intestinal epithelial cells, skin keratinocytes, and fibroblasts [259,260,261]. Thereby, exposure of TSLP stimulates CD4+ T helper type 2 differentiation [262,263]. Patients suffering from AD showed elevated expression levels of TSLP in keratinocyte, whereas healthy individuals were lacking TSLP [260,264]. Furthermore, it was demonstrated that TSLP evokes itch via TRPA1 channel activation in mice and human cell lines, thus rendering TSLP induced itch signaling histamine independent [255] (Table 1). Studies in human tissue confirmed the role of TSLP as a “missing link” between itch and AD. Hence, elevated ΔNp73 levels increased releasing of TSLP via NF-κβ activation [265]. Taken together, there is strong evidence that TSLP acts as a primary pruritogen and not as a modulator of pruritus unlike the majority of interleukins.

Altogether, there is convincing evidence for cytokines IL-31 and TSLP to play a primary role in eliciting itch sensation in humans. Moreover, other cytokines mentioned in this review and elsewhere might have a limited effect on pruritus and thus acting predominantly as modulators of itch sensation but not as primary pruritogens. In addition, a tremendous amount of experiments and research is carried out on rodent models which provides a first and strong basis for determination of the pathophysiology of itch, but ultimately the proof of concept in patients is still required. As stated by Storan and O’Gorman et al., there is convincing evidence for the important role of several interleukins in pruritus, while for cytokines such as oncostatin M, IL-2, IL-6, IL-8, and IL13R, direct evidence is still lacking [266]. This clearly demonstrates that major effort has to be invested to determine the relevance of interleukins in itch sensation in humans.

#### 3.12.2. Toll-Like Receptors

In humans, the toll-like receptor (TLRs) family is comprised of ten receptors TLR1 to TLR10, which are close homologs of the interleukin-1 receptor family [267]. Thereby, all TLR members are required to form hetero- or homo- dimers to create a functional receptor entity. This dimeric entity consists of two bitopic membrane proteins allowing for signal transduction across the membrane. TLRs regulate the host immune response against pathogens by recognizing molecular components derived from microorganisms [268]. Due to their function, TLRs are predominantly found in macrophages or dendritic cells and are activated by an enormous variety of ligands. These ligands range from lipopeptides, glycolipids, proteolipds, lipopolysaccharide, DNA, to single and double stranded RNA [269]. All TLRs, except TLR3 signal via myeloid differentiation primary response 88 (MyD88)-dependent pathways which subsequently activate transcription factor NF-κβ and Mitogen-activated protein kinase (MAPK) [268] (Table 2). Nevertheless, TLR3 signals through a TIR-domain-containing adapter-inducing interferon-β (TRIF)-dependent pathway leading to production of interferon type I. Ultimately, activation of NF-κβ leads to a similar outcome as the activation pathway of MyD88 [270] (Table 2). Additionally, TLR4 is able to signal through both TIRF- and MyD88- dependent pathways, which renders TLR4 a favored target for therapeutics [271,272] (Table 2). Recent research revealed that TLR3, TLR4, and TLR7 are expressed in small-sized pruriceptive/nociceptive neurons. Thus, there is strong evidence that these three TLRs play a potential role in mediating and modulating pruritus [273].

TLR3 interacts with exogenous double stranded RNA. This interaction leads to the production of Type I interferons, pro-inflammatory cytokines, and subsequent activation of NF-κβ [270,274,275]. It was shown that activation of TLR3 elicits an action potential in DRG neurons expressing TRPV1 and gastrin-releasing peptide (GRP). In addition, knockout and knockdown of TLR3 resulted in modulation of itch-evoked scratching behavior in mice [276]. Moreover, a study carried out on human keratinocytes and mouse DRG demonstrated that the expression of TSLP and endothelin-1 (ET-1) is increased upon exposure of polyinosinic:polycytidylic acid (PIC) [277]. PIC is a synthetic RNA homolog and acts as TLR3 agonist. In conclusion, increased levels of TLR3, TSLP and ET-1 in dry skin support the previous findings, where activation of TLR3 by PIC showed elevated itch sensation in mice. Conversely, elevated TSLP expression by TLR3 could indicate that itch sensation is evoked by direct interaction between TSLP and TRPA1 as mentioned above (Table 1 and Figure 3). Thus, rendering TLR3 an itch modulator.

TLR4 resides in the cell membrane of DRG neurons unlike TLR3 and TLR7 which are localized in the membrane of endosomes and the endoplasmic reticulum [278]. Bacterial lipopolysaccharides are the exogenous ligands of TLR4 [279]. An extensive review about synthetic and natural ligands of TLR4 from Peri et al. provides an intriguing insight into the versatility of the exogenous and endogenous ligands of TLR4 [280]. DRG sensory neurons which express TLR4 also express TRPV1 and activation of TLR4 sensitizes TRPV1 [281]. Thus, activation of these TLR4 does not elicit an acute itch sensation in mice but modulates the histaminergic itch response [282]. This research in mice supports that TLR4 enhances the histaminergic itch response by potentiating TRPV1 signaling [281,282] (Table 2). Further, studies in mice revealed that acute itch induced by acetone and diethylether followed by water was reduced upon inhibition or knockout of TLR4 [283]. In conclusion, TLR4 unlike TLR3 and TLR7, fails to evoke acute itch sensation upon activation and therefore belongs to the histamine dependent itch modulators. We have to bear in mind that the here mentioned studies about TLR4 were carried out in mice. Moreover, there is 103 order difference in LPS sensitivity between human and mice [279]. Thus, itch related insights derived from rodent experiment have to be carefully assessed before applied to humans.

Similar to TLR3, TLR7 is activated by the interaction with single stranded RNA, obviously recognizing infections by viral genomes [274,284]. Again, similar to TLR3, small-size DRG neurons which express TLR7 also express gastrin-releasing peptide, MRGPRA3 and TRPV1 in mice [285,286]. Thereby, TLR7 knockout mice did not show any impairment in pain and thermal sensitivity, or histaminergic induced itch, suggesting that TLR7 mediates histamine independent pruritus [286]. In addition, imiquimod is used to reduce growth of warts, keratosis, and basal cell carcinoma. A major side effect of the topical administered drug is itching, supporting the experimental results derived from mice [287]. Research with the TLR7 agonist imiquimod confirmed a TRPV1 dependent pathway evoking itch but proposed an TLR7 independent pathway [288] (Table 2). Altogether, there is yet little and contradicting evidence for the role of TRL7 in pruritus. Thus, major effort is required to understand the exact role of TLR3, TLR4, and TLR7 in pruritus. Moreover, results obtained in rodents have to be complemented with data derived from human cells or patients to provide a detailed and profound understanding of the role of TLRs in pruritus.

## 4. Conclusions

Besides classic chemical receptors and pathways described in this review, there are reports of a new class of pruriceptors emerging on the horizon. Mechanosensitive pruriceptors are involved in a mechanical induced itch sensation. This touch induced itch sensation involves fading of mechanosensitive Merkel cells with advancing age (alloknesis) [380,381]. Recently, it was shown that the lack of Piezo2 channel signaling (modulator) in Merkel cells is responsible for the conversion of touch into itch sensation [382]. Similarly, TLR5 positive mechanoreceptors might mediate mechanical itch conditions, because of lacking inhibition (modulation) by interneurons expressing neuropeptide Y [383,384,385]. In addition, a recent study revealed that mechanical itch was developed in streptozotocin induced diabetic mice by activation of TRPA1 [386], thus providing an explanation for itch and hypoalgesia in type 1 diabetes [386]. Altogether, mechanosensitive cells, their receptors, and signaling pathways are a potential source for itch sensation, which urgently requires future research to understand their contribution to pruritus. Thus, revealing the detailed interaction between chemical and mechanical itch pathway is of paramount interest.

Considering chemical receptors, pruritoceptive (dermal) itch is mediated through various individual receptors which interact with different mediators and modulators. Moreover, it was demonstrated that expression levels and clustering of different receptors within a cell influence itch perception. Therefore, this sensation ought to be considered as a complex interplay of different players as found in a well-rehearsed orchestra. Here, we differentiated by itch mediator and modulator. It was found that many of the presented ligands are itch modulators and do not act as primary pruritogens (Table 1 and Figure 3). As in other research fields, the majority of experiments concerning pruritus are carried out in rodents. However, the translation of results obtained from rodents cannot be applied directly to humans. Therefore, results related to itch sensation obtained in rodents have to be considered with great caution if used to decipher human pruritus. Further, the reports should be carefully consulted, and experiments have to be wisely selected to draw valid conclusions regarding the contribution of a specific receptors and their ligands to itch sensation. In addition, itch sensation is frequently accompanied by inflammation and sensations like pain or local heat [19]. These circumstances render the topic of pruritus especially challenging because of the lack of delineated boundaries between these states and sometimes even shared receptors and mediators. As is often the case, the whole is greater than the sum of its parts, and this sentence holds especially true when trying to understand the molecular mechanism of pruritoceptive itch. Nevertheless, remarkable effort and advances in the field of pruritoceptive itch have provided a multitude of new potential targets. Future research has to delineate the interaction between these targets and find ways to inhibit and modulate this sensation by targeting a combination of receptors, mediators, and modulators within the orchestra of itch.

## Figures and Tables

**Table 1:** Endogenous pruritogens and their signaling pathway. The receptors are grouped by ion channel signaling, G- protein signaling, and kinase signaling. In addition, each receptor and its endogenous pruritogen is categorized as mediator (ME) or modulator (MO). Color coded according to Figure 3.

	**ME**	**ME**	**ME**	**ME**	**MO**	**MO**	**MO**	**MO**	**MO**	**ME**	**ME**	**ME**	**ME**	**MO**	**MO**
**endogenous pruritogens**	Thrombin	Trypsin	Thrombin	Bradykinin, kallidin	Leukotriene B_4_	Leukotriene B_4_	Bradykinin, kallidin	IL-4	IL-13	IL-31	IL-2	Serotonin	BAM8-22	Calcitonin gene-related peptide	TSLP
**receptor**	PAR1	PAR2	PAR4	BDKRB1	BLT_1_	BLT_2_	BDKRB2	IL4R	IL13R	IL31R	IL2R	5-HTR7	MRGPRX1	CGRP	TSLPR
**receptor signaling**	G_i_/G_o_, G_q_/G_11_, G_12_/G_13_	G_i_/G_o_, G_12_/G_13_		G_i_/G_o_, G_q_/G_11_	G_i_/G_o_		G_s_, G_i_/G_o_, G_q_/G_11_	JAK1/2/3	JAK1	JAK1/2	JAK1/3	G_s_, G_q_/G_11_, G_12_/G_13_		G_s_	JAK1/2
**ion channel**	TRPA1, TRPV1										-	TRPA1			
	**ME**	**MO**	**MO**	**MO**	**MO**		**ME**	**ME**		**MO**	**MO**	**MO**		**MO**	**MO**
**endogenous pruritogens**	endorphins	dynorphins and endorphines	endocanna-binoids	endocanna-binoids	platelet-activating factor		substance P	Histamine		Oncostatin M	ds RNA	LPS		ss RNA	Serotonin
**receptor**	OPRM	OPRK	CB1	CB2	PAFR		NK_1_R	H_1_R		OSMR	TLR3	TLR4		TLR7	5-HTR2
**receptor signaling**	G_i_/G_o_	G_i_/G_o_, G_12_/G_13_			G_s_, G_i_/G_o_, G_q_/G_11_		G_s_, G_q_/G_11_	G_i_/G_o_, G_q_/G_11_		JAK1/2	TRIF	MyD88/TRIF		MyD88	G_i_/G_o_, G_q_/G_11_
**ion channel**	TRPV1														TRPV4

**Table 2:** Exogenous and endogenous pruritogens, their receptors, expression levels and signaling.

Receptors	Expression	G-Protein Binding [289]				Endogenous Pruritogens	Exogenous Puritogens
		G_s_	G_i_/G_o_	G_q_/G_11_	G_12_/G_13_		
		5|18	14|18	10|18	6|18		
**Histamine receptor**	**GPCR**					**Monoamine**	
H_1_R	Keratinocytes [290], dermal fibroblasts [291], Granulocytes, mast cells [292,293,294] smooth muscles, endothelial cells, CNS, and cardiovascular system [295]					Histamine [296]	vilazodone [297]
**Serotonin receptor**	**GPCRs**					**Monoamine**	
5-HTR2	Keratinocytes [298], dermal fibroblasts [299], dermal langerhans cells [74] T cells, B cells, granulocytes, dendritic cells, macrophages, and monocytes [300,301]					5-hydroxytryptamine [302]	alpha-methylserotonin [67]
5-HTR7	Keratinocytes [298], dermal fibroblasts [299], dermal Langerhans cells [74], T cells, B cells, granulocytes, macrophages, monocytes, and dendritic cells [301]					5-hydroxytryptamine [303]	LP44 [69]
**Protease-activated receptors**	**GPCRs**					**Proteases**	
PAR1	Keratinocytes [304], dermal fibroblast [305], platelets, and vascular endothelial cells [306]					Thrombin [83]	Hexapeptide derived from thedered peptide sequence e.g., TFLLR [85] bromelain, ficin, papain, mucunain, and trypsin from animals [91]
PAR2	Keratinocytes [98], dermal fibroblast [307], endothelial cells in pancreas, liver, kidney, GI tract, and colon but not in the brain or skeletal muscle [306,308]					Trypsin [83]	Hexapeptide derived from thedered peptide sequence e.g., SLIGRL [85] bromelain, ficin, papain, mucunain, and trypsin from animals [91]
PAR4	Platelets, lung, thyroid, testis, small intestin, and pancreas [306,309]					Thrombin [83]	Hexapeptide derived from thedered peptide sequence e.g., AYPGKF [85] bromelain, ficin, papain, mucunain, and trypsin from animals [91]
**Neurokinin receptors**	**GPCR**					**Peptides**	
NK_1_R	Keratinocytes, dermal fibroblast [310], dermal Langerhans cells [311], T cells, macrophages, and monocytes [312,313]					substance P, neurokinin A, neurokinin B, neuropeptide-γ, neuropeptide K [314,315,316]	N/A
**Bradykinin receptors**	**GPCRs**					**Peptides**	
BDKRB1	Keratinocytes [317], dermal fibroblasts [318], Granulocytes, and T lymphocytes [319]					bradykinin, kallidin, T-kinin, [des-Arg^10^] kallidin [320,321,322]	diphenylcyclopropenone [123]
BDKRB2	N/A					bradykinin, kallidin, T-kinin, [des-Arg^10^] kallidin [320,321,322]	sodium deoxycholic acid [117]
**Calcitonin receptor-like receptor**	**GPCR receptors**					**Peptides**	
CGRP	Keratinocytes [323,324], dermal fibroblasts [325], Lung, uterus, and placenta [326]					adrenomedullin, adrenomedullin 2/intermedin, α-CGRP, β-CGRP [327,328,329]	[Cys(Et)2,7]α-CGRP [330]
**Mas-related GPR family**	**GPCR**					**Peptide**	
MRGPRX1	Small dorsal root, and trigeminal sensory neurons [144]					BAM8-22 [331]	chloroquine [21]
**Leukotriene receptors**	**GPCRs**					**Leukotrienes**	
BLT_1_	Keratinocytes [332], Granulocytes, B-lymphocytes, leukocytes, endothelial cells, and dendritic cells [333,334,335,336]					20-hydroxy-LTB4, 12R-HETE, Leukotriene B_4_ [337,338]	oxazolone [339]
BLT_2_	Keratinocytes [340,341], spleen, liver, ovary, leukocytes, and atherosclerotic lesions [333,338]					20-hydroxy-LTB4, 12R-HETE, Leukotriene B_4_ [337,338]	oxazolone [339]
**Phospholipid receptor**	**GPCR**					**Phospholipid**	
PAFR	Keratinocytes [342], leukocytes, and granulocytes [343,344]					platelet-activating factor, methylcarbamyl PAF [345]	N/A
**Opoid receptors**	**GPCRs**					**Opioids**	
OPRM	Keratinocytes [346], dermal fibroblasts [347], CNS, T/B lymphocytes, CD4+, monocytes, macrophages, and neutrophils [348]					β-endorphin, enkephalin, dynorphin [349,350]	morphine [351]
OPRK	Keratinocytes [352], dermal fibroblasts [353], CNS, and immune cells [353,354,355]					dynorphin, β-endorphin, enkephalin, neoendorphin [349,356]	nor-binaltorphimine [357]
**Cannabinoid receptors**	**GPCRs**					**Cannabinoids**	
CB1	Keratinocytes [358], CNS, and peripheral neurons [359]					anandamide, 2-arachidonoylglycerol [360,361]	Rimonabant [362]
CB2	Keratinocytes [358], spleen, tonsils, bone marrow, and peripheral blood leukocytes [189]					anandamide, 2-arachidonoylglycerol [360,361]	N/A
**Interleukin receptors**	**non-GPCRs**					**Interleukins**	
IL2R	CD8 ^+^ T cells, and natural killer cells [363]		JAK1/3			IL-2	Aldesleukin [215]
IL4R	Keratinocytes [364], activated T-cells, and hematopoietic immune cells [365]		JAK1/2/3			IL-4	N/A
IL13R	B-cells, T-cells, basophils, eosinophils, mast cells and endothelial cells of heart, liver, skeletal muscle and ovary [366]		JAK1			IL-13	N/A
IL31R	CD14-, CD56- positive blood cells, macrophages, keratinocytes, dorsal root ganglia neurons, lung epithelial cells [241,367,368,369]		JAK1/2			IL-31	N/A
OSMR	keratinocytes, neural cells, fibroblast, and epithelial cells [367,370]		JAK1/2			Oncostatin M	N/A
TSLPR	keratinocytes [371], B-cells, T-cells, and macrophage cell lines [372]		JAK1/2			Thymic stromal lymphopoietin (TSLP)	N/A
**Toll-like receptors**	**non-GPCRs**					**various**	
TLR3	keratinocytes [373], dermal langerhans cells [374], dendritic cells, astrocytes, glia and neurons; especially in placenta and pancreas [375]		TRIF			ds RNA	Polyinosinic:polycytidylic acid [105]
TLR4	Keratinocytes [373], monocytes, macrophages, dendritic cells, and T-cells [376,377]		MyD88/TRIF			LPS	paclitaxel [378]
TLR7	Plasmacytoid dendritic cells, and B cells [379]		MyD88			ss RNA	Imiquimod [288]

## Abbreviations

AEA	arachidonoylethanolamide
BAM	bovine adrenal medulla
BAM8-22	bovine adrenal medulla peptide (8-22)
BLT_1_, _2_	leukotriene B4 receptor 1 or 2
BDK	bradykinin
BDKRB1	bradykinin receptors 1 or 2
CALCRL	calcitonin gene-related peptide type 1 receptor
CB	Cannabinoid
CB1	Cannabinoid receptor 1 or 2
CFA	Complete Freund’s adjuvant
CNS	Central nervous system
CGPR	Calcitonin gene-related peptide
eCB	Endocannabinoids
ET-1	Endothelin-1
GRP	Gastrin-releasing peptide
GRPR	Gastrin-releasing peptide receptor
GPCR	G protein coupled receptor
H_1-4_R	Histamine receptors 1-4
IL1-14	Interleukins 1-14
IL1 – 14R	Interleukin 1-14 receptor
IP3	Inositol 1,4,5 triphosphate
OPRK	κ- opioid receptor
LT	Leukotriene
LTB4	Leukotriene B4
MAPK	Mitogen-activated protein kinase
ME	mediator
MRGPRX1	Mas-related GPR family member X1
MO	Modulator
MyD88	Myeloid differentiation primary response 88
OPRM	μ- opioid receptor
OSMR	Oncostatin M receptor
PAR	Protease-activated receptors
PAFR	Platelet-activating factor receptor
PIC	Polyinosinic:polycytidylic acid
PLCB	Phospholipase CB
RAMP1	Receptor activity-modifying protein
SP	Substance P
TLR	Toll-like receptor
TRIF	TIR-domain-containing adapter-inducing interferon-β
TRPA1	Transient receptor potential cation channel, subfamily A, member 1
TRPV1, 3	Transient Receptor Potential Vanilloid 1 or 3
TSLP	Thymic stromal lymphopoietin
TSLPR	Thymic stromal lymphopoietin receptor
2-AG	2-Arachidonoylglycerol
5-HTR	5-Hydroxytryptamine receptor (serotonin receptor)
